# A pilot protocol to assess the feasibility of a virtual multiple crossover, randomized controlled trial design using methylphenidate in mild cognitive impairment

**DOI:** 10.1186/s13063-020-04752-x

**Published:** 2020-12-11

**Authors:** Libby A. DesRuisseaux, Victoria J. Williams, Alison J. McManus, Anoopum S. Gupta, Becky C. Carlyle, Hamed Azami, Jessica A. Gerber, Anna M. Bolling, Carolyn L. Cook, Rebecca A. Betensky, Steven E. Arnold

**Affiliations:** 1 MGH Interdisciplinary Brain Center, Department of Neurology, Massachusetts General Hospital, Boston, MA USA; 2 University of Heidelberg, Heidelberg, Baden Württemberg Germany; 3 College of Public Global Health, Department of Biostatistics, New York University, New York, NY USA

**Keywords:** Multiple crossover trial, Alzheimer’s disease, Mild cognitive impairment, Clinical trial design, Methylphenidate, Virtual trial

## Abstract

**Background:**

The conventional clinical trial design in Alzheimer’s disease (AD) and AD-related disorders (ADRDs) is the parallel-group randomized controlled trial. However, in heterogeneous disorders like AD/ADRDs, this design requires large sample sizes to detect meaningful effects in an “average” patient. They are very costly and, despite many attempts, have not yielded new treatments for many years. An alternative, the multi-crossover, randomized control trial (MCRCT) is a design in which each patient serves as their own control across successive, randomized blocks of active treatment and placebo. This design overcomes many limitations of parallel-group trials, yielding an unbiased assessment of treatment effect at the individual level (“N-of-1”) regardless of unique patient characteristics. The goal of the present study is to pilot a MCRCT of a potential symptomatic treatment, methylphenidate, for mild-stage AD/ADRDs, testing feasibility and compliance of participants in this design and efficacy of the drug using both standard and novel outcome measures suited for this design.

**Methods:**

Ten participants with mild cognitive impairment or mild-stage dementia due to AD/ADRDs will undergo a 4-week lead-in period followed by three, month-long treatment blocks (2 weeks of treatment with methylphenidate, 2 weeks placebo in random order). This trial will be conducted entirely virtually with an optional in-person screening visit. The primary outcome of interest is feasibility as measured by compliance and retention, with secondary and exploratory outcomes including cognition as measured by neuropsychological assessment at the end of each treatment period and daily brain games played throughout the study, actigraphy, and neuropsychiatric and functional assessments.

**Discussion:**

This pilot study will gauge the feasibility of conducting a virtual MCRCT for symptomatic treatment in early AD/ADRD. It will also compare home-based daily brain games with standard neuropsychological measures within a clinical trial for AD/ADRD. Particular attention will be paid to compliance, tolerability of drug and participation, learning effects, trends and stability of daily measures across blocks, medication carryover effects, and correlations between standard and brief daily assessments. These data will provide guidance for more efficient trial design and the use of potentially more robust, ecological outcome measures in AD/ADRD research.

**Trial registration:**

ClinicalTrials.gov, NCT03811847. Registered on 21 January 2019.

## Administrative information

Note: the numbers in curly brackets in this protocol refer to SPIRIT checklist item numbers. The order of the items has been modified to group similar items (see http://www.equator-network.org/reporting-guidelines/spirit-2013-statement-defining-standard-protocol-items-for-clinical-trials/).

**Table:** 

Title {1}	A pilot protocol to assess the feasibility of a virtual multiple crossover, randomized controlled trial design using methylphenidate in mild cognitive impairment
Trial registration {2a and 2b}.	Clinicaltrials.gov, NCT03811847 https://clinicaltrials.gov/ct2/show/NCT03811847?term=Steven+E.+Arnold&cond=Alzheimer+Disease&draw=2&rank=1
Protocol version {3}	Version 5.3 Date: August 31, 2020
Funding {4}	Alzheimer’s Clinical & Translational Research Unit through philanthropic support by the Challenger Foundation
Author details {5a}	^1^MGH Interdisciplinary Brain Center and the Department of Neurology, Massachusetts General Hospital, MA, United States of America, ^2^University of Heidelberg, Heidelberg, Baden Württemberg Germany ^3^College of Public Global Health, Department of Biostatistics, New York University, New York, NY, United States of America
Name and contact information for the trial sponsor {5b}	Alzheimer’s Clinical & Translational Research Unit actru@partners.org 617-643-2351
Role of sponsor {5c}	N/A This study is investigator-sponsored

## Introduction

### Background and rationale {6a}

Alzheimer’s disease (AD) and AD-related disorders (ADRDs) are heterogenous diseases with multiple pathophysiological processes contributing to neurodegeneration and wide-ranging clinical manifestations [1, 2]. Patients affected by these diseases often have diverse risk factors, frequent medical comorbidities, and an array of concomitant medications [3]. To accommodate these heterogeneities, conventional parallel-group randomized controlled trials (RCTs) narrow their eligibility criteria for a purer sample through various stratification strategies and increase their sample size to detect a statistically significant drug signal though the noise. This results in very large and costly multi-site studies with high screen failure rates and limited generalizability due to the selective participant sample. These factors create a nearly insuperable obstacle for many clinical trials in AD. Alternative clinical trial designs that may be more efficient include single crossover [4], multi-crossover [3], and a wide variety of adaptive design trial platforms [5]. Here, we consider a multi-crossover randomized controlled trial (MCRCT) design for a symptomatic treatment of mild cognitive impairment (MCI) or mild-stage dementia due to AD/ADRDs in which we evaluate the feasibility of the MCRCT design and compare the utility of novel outcomes suited to this design versus standard measures commonly used in typical clinical trials.

#### Multi-crossover randomized controlled trials

In MCRCTs, participants progress through multiple randomized blocks of experimental treatment and placebo or another comparator (see Jones and Kenward [4] for a detailed discussion of the design and analysis of crossover trials and Arnold and Betensky [3] for a discussion of MCRCTs in AD). Each block consists of an experimental treatment period “A” and a placebo treatment period “B” that are administered in a randomized order [4]. The participant goes through multiple iterations of these blocks throughout the trial (e.g., AB-BA-AB) with or without washout periods between each block. Because each participant serves as their own control in the MCRCT design, most factors that unbalance comparison groups in a parallel design are eliminated [6, 7]. Further, this design is able to measure the efficacy of a given intervention in the context of unique individual characteristics (“N-of-1”), facilitating a personalized medicine approach [6, 7]. However, when a collection of MCRCTs implement the same design, treatment, and outcome measures across participants, data can also be easily combined to gauge a treatment’s efficacy for the population of interest in a manner similar to the generalizability of effects inferred from traditional parallel-group RCTs [7].

Important elements of a MCRCT include block repetition, randomized block sequence assignment, blinding, and systematic outcome measures [3]. With repetition and block randomization, the effects of random or systematic events on outcome measures are attenuated, such as those due to life events or disease progression. The randomization of the treatment block sequences also reduces time-dependent confounders, such as treatment carryover effects while blinding raters to reduce potential bias in the assessments of outcome measures. The three-block MCRCT design overcomes a major disadvantage of a single crossover design by allowing for within-subject analyses of carryover effects and the direct treatment interaction [4]. Although more block repetitions afford greater statistical power, factors such as trial duration, participant burden and retention, cost, and progression of disease course must also be considered. Therefore, we determined that a three-block, 16-week study design would be best for this pilot trial.

Special consideration is needed in the selection of treatments and outcome measures for MCRCTs. Given the multiple crossovers, MCRCTs are best for drugs or other treatments with rapid onsets and cessations of action in order to reasonably minimize the duration of each treatment and placebo period and minimize residual pharmacological effects after crossovers. Although MCRCTs can be designed to employ a range of customizable outcome measures, these measures must be able to accommodate repeat administration and potential acclimatization effects. Frequent or even daily assessments are especially well-suited as outcome measures for this trial design. With repeat exposure and familiarization with assessments over time, practice effects will decline, and the participant’s learning curve will plateau. Once this point has been achieved, performance variations are more likely the result of fluctuations in cognitive performance rather than learning or practice effects.

#### Daily assessments

The day-to-day variability of cognitive functioning in AD/ADRDs is a challenge faced by many clinical trials. In conventional trials, major outcome measures are acquired at the beginning and end of the trial and sometimes at periodic assessments along the way (e.g., every 3 or 6 months to observe time course). At the trial’s conclusion, each participant has only a handful of data points with which the efficacy of the drug will be determined. Cognitive performance is subject to many sources of variation such as neuropsychiatric symptoms [8], the duration and quality of sleep obtained the night before testing [9, 10], the experience of recent physical pain [10], and the ill-defined “good days and bad days” of MCI and dementia. Studies have even posited that the degree of intraindividual variation between testing sessions may serve as a marker of cognitive impairment and future decline in and of itself [11, 12]. Such variation cannot be appreciated with infrequent measures. The repetition in MCRCTs is particularly well-suited for employing customizable daily assessments to increase confidence in capturing the participant’s true changes in cognition through tracking and averaging measures with more frequent assessment, while also apprehending observable patterns of fluctuating performance as an additional endpoint.

#### Other functional, neurophysiological, and neuropsychiatric measures in ADRD

Dementias are defined by cognitive deficits, but other aspects of brain function are also affected in AD/ADRDs. Impairments in movement, sleep, and neuropsychiatric symptoms are common [13] and can be more disabling or distressing than cognitive impairments. As with cognitive deficits, these symptoms are also variable between and within individuals, and the repetition of MCRCTs allows for a more robust comparison of these non-cognitive features between treatment conditions at the individual level.

Several exploratory assessments of noncognitive domains will be implemented in this trial, including the use of daily, home-based assessments. MCRCTs lend themselves well to daily measurement with wearable technologies to monitor physical activity level as well as simple, daily subjective ratings of emotion, behavior, and well-being as people log on to play their brain games. MCRCTs with innovative, repeatable digital measures and daily phenotyping allow for a more comprehensive and efficient analysis of treatment efficacy.

#### Virtual trial conduct

Due to the COVID-19 pandemic, its uncertain course, and the particular vulnerability of the elderly participant population, this trial will be conducted entirely virtually. All study visits will occur via a HIPAA-compliant videoconferencing platform. However, participants will be given the option to come into the clinical research center for their screening visit if they elect to do so. All outcome measures will be collected virtually.

### Objectives {7}

The primary objective of this clinical trial is to pilot the feasibility of a virtual MCRCT design in approximately 10 participants with MCI or mild-stage dementia due to AD/ADRD. Secondary objectives will examine the efficacy of MPH on cognition as measured by daily cognitive assessments compared to more standard single time-point neuropsychological outcome measures. The exploratory objectives are to investigate the effects of MPH compared to placebo on neuropsychiatric and daily functioning with standard measures, home-based actigraphy, and other novel digital cognitive and neurophysiological assessment. Analyses will primarily focus on within-participant comparisons. Due to the small sample size of this pilot, between-participant comparisons will be exploratory and findings will be used to inform the design of larger MCRCTs.

### Trial design {8}

**Fig. 1:**
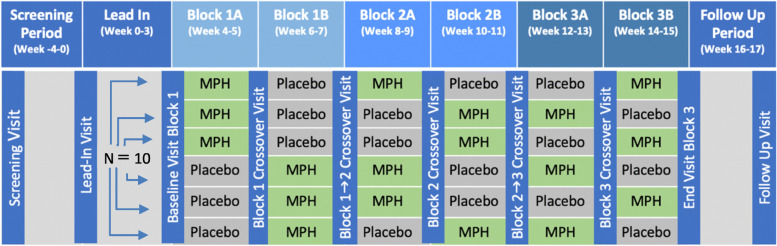
Trial design schema. Each participant will be randomized into one of six block randomization sequences such that each sequence will have at least one participant assigned to it

This is a virtual multi-crossover, double-blind, placebo-controlled randomized sequence block trial. Each volunteer will engage in a four-week placebo lead-in and acclimation period followed by three MPH versus placebo crossover “blocks” (see Fig. 1). Each four-week block will consist of two weeks of active treatment and two weeks of placebo. There are eight possible sequence orders in a three-block MCRCT; however, we will use only six (Fig. 1) to ensure some counterbalancing of randomization order among the blocks and avoid the two sequence orders in which a systematic sequence bias exists (AB-AB-AB and BA-BA-BA).

## Methods: participants, interventions, and outcomes

### Study setting {9}

The single site for this study will be Massachusetts General Hospital in Boston, MA, USA.

### Eligibility criteria {10}

Complete eligibility criteria are described in Table 1.

**Table 1:** Inclusion/exclusion criteria

Inclusion criteria	Exclusion criteria
1. Male or female, aged 55–95 inclusive 2. Diagnosis of MCI or mild-stage dementia presumed due to AD and AD-related disorders 3. Cognitive abilities sufficient to be able to complete all study tasks as determined by the PI or a co-investigator 4. Education level, English language skills, and literacy that indicates participant will be able to comprehend all assessments 5. Neuropsychiatric Inventory Agitation/Aggression Question 4 = “No” or “Yes” with a mild severity rating 6. Willing and able to complete all assessments and study procedures 7. Not pregnant, lactating, or of child-bearing potential 8. Volunteer has a study partner with at least 2 days per week of contact and willing to complete partner study forms 9. No exclusionary medications or dietary supplements. See Section 6.5.8.1 10. If on cholinesterase inhibitor and/or memantine, doses are stable for 3 months prior to baseline 11. Basic videoconferencing capabilities and a willingness to participate in a virtual trial (including self-administered ECG)	1. Any history of specific central nervous system disease other than AD/ADRDs, such as major clinical stroke, brain tumor, normal pressure hydrocephalus, multiple sclerosis, significant head trauma with persistent neurological or cognitive deficits or complaints 2. Clinically significant or unstable medical condition that could affect safety or compliance with the study and would, in the opinion of the investigator, pose a risk to the participant if they were to participate in the study 3. Major active or chronic severe psychiatric illness (e.g., major depression, bipolar disorder, obsessive compulsive disorder, schizophrenia) within the previous year 4. Current suicidal ideation or history of suicide attempt 5. History of alcohol or other substance abuse or dependence with the past 2 years 6. Clinically significant abnormalities on complete blood count, comprehensive metabolic panel, vitamin B12, or thyroid-stimulating hormone screening safety lab results 7. Concomitant use of medications with psychoactive properties that may deleteriously affect cognition (anticholinergics, antihistamines, antipsychotics, sedative hypnotics, anxiolytics) 8. Treatment with monoamine oxidase inhibitors, coumadin, phenobarbital, phenytoin, primidone, tricyclic antidepressants, or other medicines with potential for clinically significant interaction 9. Hypersensitivity to MPH 10. History of marked anxiety and agitation, ADHD, motor tics, glaucoma, or a history or family history of Tourette’s syndrome 11. Clinically significant cardiac condition for which MPH may be contraindicated as determined by study physician, such as MI or ventricular arrhythmia within 6 months of enrollment 12. History of untreated, uncontrolled hypertension or a blood pressure greater than 150/90 at screening visit 13. Use of other small molecule or device-based investigational agents 1 month prior to entry and for the duration of the trial

### Who will take informed consent? {26a}

Informed consent will be obtained at the screening visit by a physician or nurse practitioner. Virtual screening visits will occur via a secure videoconferencing platform and contain all elements of a typical informed consent process. The virtual consent procedure will be performed according to the recommendations of the local Institutional Review Board. The informed consent process will be conducted using a REDCap-based electronic consent form. During the screening visit, the participant and study partner will be emailed a link to the REDCap e-consent form and the document will be reviewed with the participant and study partner by a licensed clinician investigator. The virtual consent process will also involve the participation of a subject advocate, a healthcare worker who is not a member of the study team, who will sign the consent form to attest that the participant provided meaningful consent.

### Additional consent provisions for collection and use of participant data and biological specimens {26b}

Participants will be asked if they agree to use of their data and for permission for the research team to share relevant data with research collaborators and regulatory authorities. This trial does not involve collecting biological specimens that will be stored long term.

### Interventions

#### Explanation for the choice of comparators {6b}

As a proof of concept trial to assess MCRCT feasibility in AD/ADRDs and to evaluate symptomatic efficacy of a psychostimulant medicine on novel outcome measures, we designed a brief MCRCT using methylphenidate (MPH). MPH is a fitting drug for a brief MCRCT design due to its pharmacokinetic and pharmacodynamic profile with rapid onset and cessation of activity, which minimize concern for carryover effects when switching from treatment to placebo.

MPH is a phenethylamine/benzylpiperidine psychostimulant drug approved for treatment of attention deficit/hyperactivity disorder (ADHD). Usefulness of MPH has also been suggested for depression [14–16], neuroprotection against Parkinson’s disease [17], and general memory and attentional cognitive enhancement [18, 19]. The safety and tolerability of this drug in elderly patients with dementia is generally well-established. MPH is commonly prescribed in various neurodegenerative dementias, but cognitive and behavioral efficacy data from rigorous clinical trials are scant. Improvements in apathy have been reported in clinical trials in AD, typically in moderate or advanced stages [20–24], and to lesser degrees in other dementias [25, 26].

#### Intervention description {11a}

Participants will take MPH or matching placebo once daily for 16 weeks. An extended release formulation of methylphenidate was chosen to minimize fluctuations between peak and trough concentrations of multiple doses of immediate release MPH. Medication will be dispensed in the form of 18 mg capsules. Participants will be sent study medication at each visit from lead-in (week 0) to block 3b (week 14). Study medication will be delivered directly to the participants’ homes using a medical courier, and participants will sign for the medication upon its arrival.

In order to safely titrate participants to the 36-mg dose, participants will take 18 mg of MPH for the first week of each 2-week treatment period. If the 18-mg dose is well-tolerated, the participants’ dose will be increased to 36 mg for the second week of the treatment period. To determine safety of a dose escalation, a licensed study clinician will call the participant to determine whether the participant has experienced any clinically significant adverse effects before and after dose escalation. Because this is a double-blind study, dose escalation calls will occur during block 1a and block 1b. If the participant tolerates the 36 mg during block 1, then the participant will be escalated on blocks 2 and 3. If the participant does not tolerate the 36-mg dose, they will remain on the 18-mg dose for the duration of the trial. The 18–36-mg range is commonly used in clinical care of elderly patients. MPH will be over-encapsulated to be visually identical to placebo capsules.

#### Criteria for discontinuing or modifying allocated interventions {11b}

Reasons for discontinuation of study medication may include an adverse event, principal investigator (PI) recommendation, protocol deviation, loss-to-follow-up, patient request, or death. Study participants who discontinue study drug prematurely and decide not to remain in the study will be encouraged to return for an early discontinuation visit.

#### Strategies to improve adherence to interventions {11c}

Participants will be given the exact quantity of pills they will be expected to take between delivery and their next visit. Should there be any unused pills (due to missed doses or for a participant who did not tolerate escalation to the 36-mg dose), the medical courier will return these pills to the study team. The study team will then count and log the number of remaining capsules to determine compliance before subsequent destruction of the pills. Non-compliance will be defined as taking less than 80% or more than 125% (e.g., if the participant is not successfully escalated to the 36 mg during the first block yet takes more than the prescribed 18-mg dose) of study medication as determined by returned capsule counts. If a participant is non-compliant with study medication, research staff will re-educate the participant in administration of study drug. If the participant’s non-compliance persists, it will be left up to the PI to determine whether the participant should be discontinued from the study.

Compliance with study tasks will be assessed at each study visit and via ongoing monitoring of log-ons and daily brain game and survey completion. Participants are also provided with study calendar checklists that demonstrate when home-based tasks should be completed and allow participants to record when they have completed each task.

#### Relevant concomitant care permitted or prohibited during the trial {11d}

Concurrent treatment with contraindicated medications (e.g., monoamine oxidase inhibitors) is prohibited. Because of possible increases in blood pressure, MPH will be used cautiously with vasopressor agents. MPH may also inhibit the metabolism of coumadin, phenobarbital, phenytoin, primidone, and tricyclic antidepressants, so participants requiring these medications will be excluded.

### Provisions for post-trial care {30}

After completion of the trial, participants will receive feedback as to whether or not treatment with MPH was effective for them. These results may be communicated to their clinical care team to inform future treatment options, but there will not be any ancillary, post-trial care provided by the study team.

### Outcomes {12}

Primary outcome measures will consist of measures of feasibility including completion rates of the various outcome measures, medication compliance, study retention, qualitative participant feedback about tolerability of study tasks, and adverse events. Secondary outcomes will explore the efficacy of MPH treatment on cognitive functioning as measured by both daily measures of cognition and gold standard neuropsychological outcome measures virtually administered between treatment blocks. Exploratory outcome measures will also be assessed including adverse events, and the effects of treatment on neuropsychiatric symptoms, actigraphy, and measures of daily functioning (see Tables 2 and 3 for schedule and assessments and outcome measures).

**Table 2:**
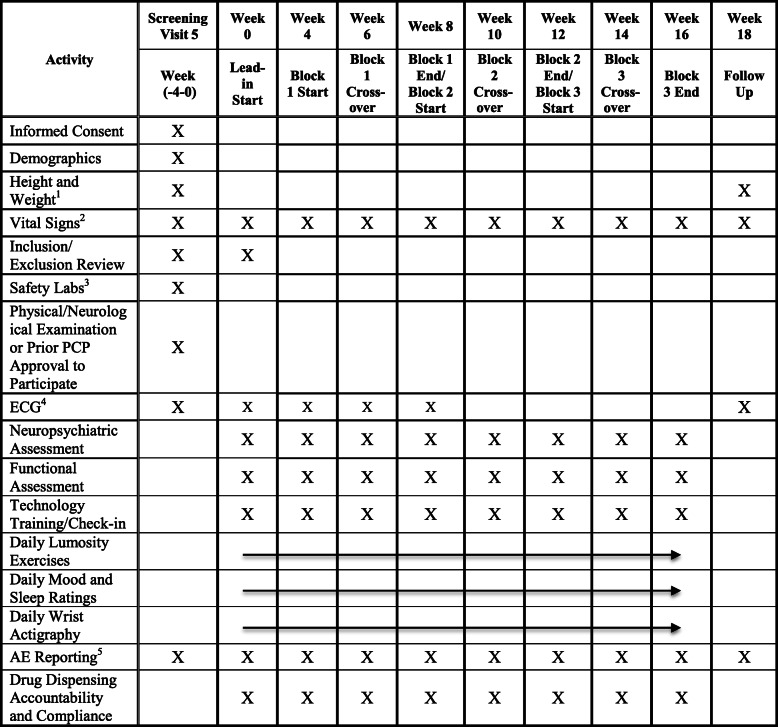
Study schedule of assessments

^1^If the screening visit does not occur in-person, the participant will self-report height and weight

^2^At-home vital signs will include blood pressure and heart rate and will be measured using an automatic blood pressure cuff that will be sent to the participant

^3^If the screening visit does not occur in-person, the participant will go to their nearest LabCorp location to have safety labs drawn

^4^At an in-person screening visit, a standard 12-lead ECG will be used. However, if the screening visit occurs virtually and for all other study visits, 6-lead ECGs will be obtained via the KardiaMobile 6L device (AliveCor, Inc)

*A virtual screening visit will occur in two parts. Part 1 will consist of informed consent, demographics, and self-report height and weight. Between parts 1 and 2, participants will go to a third party lab to obtain blood work and ask their PCP for approval to participate in the trial. The study team will also send a blood pressure cuff and the KardiaMobile 6L device to participants during this time. At part 2, the study team will review blood results with the participant and collect vital signs and ECG measurements

**Table 3:** Outcome measures grouped by domain

	Standard measures	Daily measures
**Cognitive function**	RBANS Oral Trail Making Test Continuous Performance Task BVMT-R Speech production task	Lumosity games - Speed - Memory - Problem-solving - Attention - Flexibility
**Neuropsychiatric symptoms**	Agitated Behavior in Dementia Scale Apathy Evaluation Scale Beck Anxiety Inventory Geriatric Depression Scale	Daily mood ratings Daily sleep ratings
**Functional abilities**	Functional Activities Questionnaire ADCS-ADL-MCI Dementia Severity Rating Scale	Fitbit activity tracking
**Physiological measures**	6-lead ECG using the KardiaMobile 6L device (AliveCor, Inc.)	Fitbit heart rate tracking Fitbit sleep tracking

Standard cognitive and neuropsychiatric/functional measures will be administered virtually at each study visit via a videoconference by appropriately trained study staff (neuropsychologists or psychometrists). ECG and vital signs will be collected by the participant or the study partner at home during the videoconference under the supervision of a clinical research coordinator who has been certified to measure vital signs. Blood pressure, pulse rate, and ECG readings will be reviewed by a registered nurse, nurse practitioner, or medical doctor.

### Participant timeline {13}

The study timeline is depicted in Table 2.

#### Feasibility outcomes

Primary feasibility outcomes will include measures of retention, adherence/compliance, and safety. Study protocol compliance will be based on completion rates for each of the outcome measures described in this trial, which will subsequently be combined across participants to form 95% confidence intervals for each outcome. Medication compliance will be determined based on each participant’s pill reconciliation conducted between each block, and subsequently combined across all participants to generate a measure of overall medication compliance. Study retention will be assessed using completion rates of all participants enrolled in this pilot. Finally, we will collect qualitative feedback from each participant throughout the trial and upon study completion (or early termination) to assess their opinions on the tolerability and acceptability of the MCRCT design and the various study tasks. Areas of particular interest will include duration of study participation, frequency of assessments, ability to navigate all study technology, use of the virtual platform, and overall burden of daily assessments.

#### Cognitive outcomes

##### Standard assessments

The Repeatable Battery for the Assessment of Neuropsychological Statues-Update (RBANS, [27]) will serve as the primary standard endpoint for cognition. The RBANS measures five neurocognitive domains (immediate memory, visuospatial, language, attention, and delayed memory) and has five parallel versions, making it particularly suitable for repeat assessments. The Brief Visuospatial Memory Task-Revised (BVMT-R, [28]) and the oral version of the Trail Making Test [29–31] will also be used as measures of visuospatial memory and of processing speed, mental flexibility, and set-switching, respectively. All stimuli will be presented virtually via videoconference in accordance with appropriate the publishing companies’ licensing agreements and copyright laws.

Exploratory measures include the Months Forward and Backward Test (processing speed, [32]) a narrative speech production task (speech rate and rhythm, semantic knowledge, and productive fluency), and the Continuous Performance Test (sustained attention and inhibitory control, [33]) which will be administered using Inquisit Web software (Millisecond, Inc).

##### Daily cognition

Participants will be asked to complete Lumosity brain games (Lumos Labs, Inc.) at least 6 days per week. A completely de-identified Lumosity research account will be given to each participant for use during the study. Participants will alternate between two different daily batteries of six games each that will be generated automatically on their account home screen. Games sample the cognitive domains of attention, calculation, cognitive flexibility, memory, processing speed, and problem-solving (see Table 4). Participants will be informed that the study team will be checking this account regularly to monitor their progress.

**Table 4:** Lumosity game for daily, home-based assessment of cognition

Domain	Subdomain	Battery	Game	Description
Attention	Divided attention	1	Playing Koi	Feed each fish in a moving school of fish only once
	Selective attention	2	Lost in Migration 2	Indicate the direction in which the middle bird in a flock of birds is flying (Flanker test)
Calculation	Numerical estimation	1	Chalkboard Challenge 2	Determine which of two values or equations is greater
Cognitive flexibility	Task switching	1	Ebb and Flow	Indicate the direction leaves are either pointing or moving across the screen
	Task switching	2	Brain Shift 2	Rule switching task—answer yes/no questions about letter-number pairs based on location of stimuli presented
	Response inhibition	2	Color Match 2	Stroop color-word interference task
Memory	Spatial memory	1	Memory Matrix 2	Memorize locations of tile patterns on grid
	Working memory	1	Pinball Recall	Memorize locations of bumpers on grid and use their locations to predict where a pinball will deflect after hitting the bumpers
	Working memory	2	Follow That Frog	Remember the path taken by a frog as it hops around on lily pads
Processing speed	Information processing	1	Spatial Speed Match 2	Indicate whether the symbol on the present card is in the same location as the symbol on the previous card (“1-back” task)
	Information processing	2	Speed Match 2	One-back task—indicate whether the symbol on the present card matches the symbol on the previous card (“1-back” task)
Problem-solving	Spatial reasoning	2	Masterpiece	Fit different shapes of tiles together to form a predetermined shape

Participants complete six games per day in two alternating, preset batteries, yielding three data points per game per week. Participants are allowed to repeat games in a battery. If games are repeated, the highest score for that day is selected

#### Functional outcomes

##### Standard assessments

To assess a participant’s functional abilities and offer a global characterization of everyday activities that may be impacted by neurodegenerative disease and treatment with MPH, three informant-administered scales will be administered at each block visit: the Functional Activities Questionnaire (FAQ, [34]), the Alzheimer’s Disease Cooperative Study/Activities of Daily Living scale adapted for MCI patients (ADCS-ADL-MCL, [35]), and the Dementia Severity Rating Scale (DSRS, [36]).

##### Daily outcomes

Participants will wear the Fitbit Charge 3 wrist actigraph to track their daily activity levels, including steps taken and daily active minutes.

#### Neuropsychiatric assessments

##### Standard assessments

Individuals with AD/ADRD often suffer from a range of neuropsychiatric symptoms, including apathy, depression, agitation, and irritability. Past studies have shown that MPH may alleviate [20–24] or worsen these symptoms [37]. To measure neuropsychiatric functioning and monitor treatment effects, we will use both self-report and informant-report scales, including the Geriatric Depression Scale (GDS, [38]), Apathy Evaluation Scale (AES, [39]), Beck Anxiety Inventory (BAI, [40]), and Agitated Behavior in Dementia Scale (ABID, [41]).

##### Daily outcomes

Participants will complete a daily survey before starting their Lumosity exercises that asks them to rate their mood and sleep quality.

#### Neurophysiological assessment

##### Standard assessment

To monitor cardiac safety, participants will be sent the KardiaMobile 6L device (AliveCor, Inc), a medical-grade at-home 6-lead ECG device. ECGs will be completed at each visit from the screening visit through the end of block 1 (week 8) to ensure there are no adverse cardiac effects of MPH treatment. If any clinically significant cardiac abnormalities become apparent during this time, ECGs will be completed through the end of the trial.

##### Daily outcomes

The participants’ daily heart rate and sleep will be tracked by the Fitbit Charge 3 actigraph. Heart rate data is measured as average beats per minute (BPM) across a given interval, with the smallest unit being average BPM over 1 min. Characteristics of sleep, including time spent asleep, time spent in different sleep stages, and number of awakenings, will be collected.

#### Sample size {14}

This study will randomize 10 participants with the goal of having 8 participants complete the study. The present study is a pilot study to primarily determine the feasibility of this “N-of-1” MCRCT design. Therefore, sample size determination was not calculated based on the estimated effect of the intervention [42] but was decided based on the number of participants that would allow for the assessment of feasibility and tolerability of the study design. Analysis of the other co-primary outcome, determining the effects of MPH on cognition, will be done within-subjects. Any between-subject comparisons will be purely exploratory.

#### Recruitment {15}

Participants will primarily be recruited from the Massachusetts General Hospital Memory Disorders Unit, an outpatient clinic specializing in evaluation of older adults with cognitive complaints. Subjects may also be recruited through online advertisements on Partners Healthcare Rally for Research (rally.partners.org).

### Assignment of interventions: allocation

#### Sequence generation {16a}

Six randomization sequences consisting of three crossover blocks have been generated by an off-site study biostatistician. Only adequately counterbalanced sequences will be used (i.e., AB-AB-AB and BA-BA-BA sequences will be excluded).

#### Concealment mechanism {16b}

Participants are assigned to a sequence by a non-study staff pharmacist with at least one participant per sequence (see Fig. 1). On-site study staff will not know participants’ assigned treatment sequence.

#### Implementation {16c}

Participants will be enrolled by on-site research staff. All participants will receive the same intervention and outcome measures.

### Assignment of interventions: blinding {17a-b}

#### Who will be blinded {17a}

All on-site staff, study participants, and study partners will remain blinded until the participant’s data has been locked unless unblinding is medically necessary.

#### Procedure for unblinding if needed {17b}

If unblinding is necessary, the off-site pharmacist will reveal the participant’s current treatment phase (MPH or placebo).

### Data collection and management

#### Plans for assessment and collection of outcomes {18a}

Data collection will be performed by trained study staff. Prior to the lead-in visit, the subjects will receive a package with all materials that will be necessary for the collection of outcome measures. Before each visit, subjects will also be sent any response forms for the cognitive assessments. The cognitive assessments and surveys will be completed during the videoconference. At the conclusion of the visit, participants will put all response forms in an envelope that they will then seal while on camera. Response forms will be mailed back to the study team to be scored and stored in the participant’s study binder.

Study staff will adequately train participants and their study partners on study technology and completion of daily, home-based assessment at the lead-in visit (week 0). All participants will be provided with documents thoroughly detailing how to complete all home-based study tasks. They will also be given a study compliance calendar that specifies when each home-based task should be completed. Study coordinators will conduct three follow-up phone calls with participants (day 1, week 1, and week 3) to ensure the study technology is functioning properly and that participants are able to successfully complete all home-based study tasks. Compliance will be assessed weekly by the study coordinator by logging on to each participant’s Lumosity account. Task performance data will not be accessed in this process. If the participant is at risk of not meeting their weekly study task requirements, the study coordinator will contact the participant or their study partner to remind them of study expectations. If a participant requires more than three such reminders, they will be considered non-compliant, and the PI will determine if the participant should continue in the study or if their study participation should be discontinued.

#### Plans to promote participant retention and complete follow-up {18b}

Retention is not anticipated to be a major concern as participants will have frequent contact and interaction with study staff during bi-weekly visits.

#### Data management {19}

The PI will ultimately be responsible for the validity and integrity of the data collected and for ensuring that the study is conducted in accordance with the IRB-approved protocol. Study data will be collected and managed using REDCap electronic data capture tools [43, 44] hosted by Partners Healthcare. The PI or designee will conduct monthly source data verification reviews. All electronic documentation will be stored on password-protected devices in locked cabinets located in secure areas. All paper documentation will also be stored in these locked and secure areas.

### Confidentiality {27}

All participants will receive a study identification number upon entry into the trial. This code will be used for all study tasks, including Lumosity and Fitbit accounts. The key linking participant identity to study ID will be stored in secure locations and only accessible to study staff. No personally identifying or protected health information will be used for the generation of the Lumosity or Fitbit accounts.

### Plans for collection, laboratory evaluation, and storage of biological specimens for genetic or molecular analysis in this trial/future use {33}

See item 26b—no biological specimens will be stored in this research study.

### Statistical methods

#### Statistical methods for primary and secondary outcomes {20a}

This pilot trial will help us to determine the feasibility of this MCRCT design, with secondary aims exploring the effects of MPH treatment on a novel measure of daily cognition in participants with AD/ADRD. The core data of a MCRCT are the measurements obtained in two or more different treatment conditions occurring repeatedly over time. The goal is to compare outcomes for each condition within a randomized block structure while accommodating repetition within participants and possible effects of carryover or disease progression. We will analyze all randomized participants who complete at least one block (two treatment periods). Individuals who complete only one block will be used in exploratory group-level analyses and individual-level analyses that combine multiple endpoint measures.

#### Primary outcomes

Primary feasibility outcomes of the MCRCT design will be assessed using several approaches. We will assess study protocol compliance by calculating completion rates for each of the outcome measures described in this trial, which will subsequently be combined across participants to form 95% confidence intervals for each outcome. Comparison of completion rates across outcome measures will allow us to determine which outcomes were most/least tolerated by study participants, with particular interest comparing completion rates between daily measures and more standard outcome measures that will be assessed at each crossover.

Second, we will assess study retention by comparing study completion rates of this pilot MCRCT design to previously published completion rates for more RCT designs in AD populations. A recent study comparing study retention rates of clinical trials across the ADRD spectrum found that completion rates ranged from 46 to 95% (mean = 71.6%) for MCI populations, and from 59 to 89% (mean = 77.7%) for mild to moderate AD populations [45]. Thus, a benchmark goal for study retention will be ≥ 80% retention of randomized participants. Medication compliance will be calculated based on each participant’s pill reconciliation conducted between each crossover, and subsequently combined across all participants to generate a measure of overall medication compliance.

Finally, we will collect qualitative feedback from each participant throughout the trial and upon study completion (or early termination) to assess their opinions on the tolerability and acceptability of the MCRCT design and of the various study tasks. Areas of particular interest will include duration of study participation, frequency of assessments, ability to navigate all study technology, use of the virtual platform, and overall burden of daily assessments. Adverse events will also be tracked and reported. Benchmark goals for feasibility measures are set as follows: retain ≥ 80% of participants enrolled, observe ≥ 80% medication adherence, and ≥ 80% outcome assessment completion rates.

#### Analysis of secondary and exploratory outcomes

We will have two measures for assessing the efficacy of MPH treatment on cognitive outcome: (1) the RBANS Total Score administered at baseline, each crossover, and end of study, and (2) a daily composite index of Lumosity game scores created by averaging each game’s normalized score on that day and then averaging the composite indices over the treatment period. These secondary analyses will follow the methodology outlined in Senn [7], at both the individual level, followed by exploratory analyses at the group level.

We will first analyze each individual separately, as in a true N-of-1 study. To assess data quality and distribution, we will inspect histograms and QQ plots of the MPH outcomes and of the placebo outcomes. If normality is affirmed, we will conduct a paired *t* test (pairing within each block) to formally test the equality of the MPH outcome and the placebo outcome. This amounts to calculating differences between placebo and MPH, within each block, and always in the same direction, e.g., MPH-placebo. We will calculate a 95% normal-based confidence interval for the mean difference. If there are strong deviations from normality, we will conduct an exact Wilcoxon signed rank test and report the Hodges-Lehmann estimate and its confidence interval instead. We will then combine the data across individuals and conduct the same tests and calculate the same estimates for these exploratory group-level analyses. Exploratory group-level analyses will provide us with useful estimates of within- and between-subject variances for the novel cognitive outcome measures (Lumosity), which will help inform sample size to detect group effects in subsequent trials.

Another major goal of this pilot is to compare the sensitivity of daily brain games (i.e., Lumosity) to more standard cognitive assessment (i.e., RBANS) in detecting treatment effects. To do this, a linear regression will be fitted to model the average brain games assessment scores over the treatment blocks using the RBANS scores from the end of the same block. If after performing the initial regression, a nonlinear relationship is suspected, the appropriate transformations will be applied to find the best-fitting model. A large correlation coefficient from a linear or nonlinear model will suggest that future studies could rely more heavily on daily home-based assessments, eliminating many of the constraints that apply to the current gold standard assessments that are optimally administered in a clinic/office setting.

In exploratory analyses, we will fit mixed effects regression models for the individual components of the RBANS and for the Lumosity data across time points and for the separate Lumosity tests. We will consider adjusting for the baseline levels of each test in its respective model. We will also include the treatment from the previous period as a covariate to assess carryover that may occur between and within blocks. Based on the pharmacokinetics and pharmacodynamics of MPH, we do not expect there to be any biological carryover effects. However, in order to investigate the validity of this assumption, we will assess whether a washout period for the Lumosity data is necessary through modeling the entire series of data across all time points, and formally testing for differences within treatment period between the first half and second half of the period. If we find that some washout is necessary, we will consider different lengths of washout necessary by successively testing differences between the first *X* days and the second 14-*X* days of the period and selecting the maximum *X* for which there is no meaningful difference. Each endpoint will be associated with a different magnitude for this meaningful difference.

Additional exploratory goals of this study are to assess the potential utility of other novel home-based assessments in an MCRCT for AD/ADRD. We will do so by assessing completion rates and qualitative participant feedback on the acceptability of these measures.

#### Interim analyses {21b}

No stopping guidelines are planned in this pilot study.

#### Methods for additional analyses (e.g., subgroup analyses) {20b}

No additional analyses are planned in this pilot study.

#### Methods in analysis to handle protocol non-adherence and any statistical methods to handle missing data {20c}

We do not expect any missing RBANS data from this small study. If there are any, we will assume the missingness to be completely at random and not related to the treatment or to any other factors, given the short time frame of the study. Under this assumption, we will analyze the data that are available. If an entire block is missing, we will analyze the available blocks as described for the complete data. If one 2-week period is missing, we will combine the available block with the others of the same treatment, e.g., two blocks rather than three blocks if an entire block is missing. If there are missing Lumosity data, we will also assume them to be missing completely at random and use the available data from each participant. In secondary analyses, we will closely examine whether the missing or partially missing Lumosity data may be informative and constitute a useful endpoint itself.

We will consider novel endpoints for the Lumosity data, such as the patterns of missingness. These may be informative about the difficulty experienced by the participant and thus may provide useful information regarding treatment efficacy.

Protocol non-adherence will be defined as completion rates, 75% for daily study tasks and < 80% study medication dosing. If a participant demonstrates persistent non-adherence during the study and requires frequent reminders to complete study tasks, they will be discontinued from the study

#### Plans to give access to the full protocol, participant-level data, and statistical code {31c}

The full protocol, dataset, and statistical code may be made available from the corresponding author upon request.

### Oversight and monitoring

#### Composition of the coordinating center and trial steering committee {5d}

The trial will be conducted by the Alzheimer’s Clinical & Translational Research Unit (ACTRU) at Massachusetts General Hospital directed by Dr. Steven Arnold, MD. The ACTRU research team consists of clinical research coordinators, research nurses, project and program managers, neuropsychologists, and signal processing engineers who play a role in the conduct of this trial. Potential participants are identified and screened by a research nurse. Participants are consented by a licensed physician or advanced nurse practitioner. Clinical research coordinators are also responsible for the majority of data collection. The ACTRU team meets weekly to discuss the conduct of the trial and ensure participant safety.

#### Composition of the data monitoring committee, its role and reporting structure {21a}

Data monitoring committees assess safety and treatment efficacy; however, because this is a small, single site pilot study utilizing an FDA-approved drug, a data monitoring committee is not necessary. Participants will be monitored for adverse events from the time they sign consent until completion of their participation in the study. The study procedures and the well-being of all participants will be monitored closely by the PI and the co-investigators. Throughout the course of the study, constant feedback with the participant is maintained in order to assess compliance, comfort, and safety and to minimize risks throughout the procedure.

#### Adverse event reporting and harms {22}

A study participant will be discontinued from participation in the study if any clinically significant adverse event (AE), laboratory abnormality, concurrent illness, or other medical condition or situation occurs such that continued participation in the study is not in the best interest of the participant, or the participant meets any exclusion criteria (either newly developed or not previously recognized). The primary safety endpoint will be the incidence of treatment emergent grade II–IV adverse events. Other safety endpoints include clinical laboratory tests, vital signs, physical examinations, ECGs, use of concomitant medications, and the Columbia-Suicide Severity Rating Scale (C-SSRS).

The PI or co-investigators will monitor each participant for clinical and laboratory evidence of adverse events on a routine basis throughout the study. The investigator will assess and record any adverse event in detail, including the date of onset, event diagnosis (if known) or sign/symptom, severity, time course (end date, ongoing, intermittent), relationship of the adverse event to study drug, and any action(s) taken.

#### Frequency and plans for auditing trial conduct {23}

All data entered will be reviewed by a secondary study coordinator for accuracy and completeness. The PI or an appointed designee will conduct monthly source data verification reviews.

#### Plans for communicating important protocol amendments to relevant parties (e.g., trial participants, ethical committees) {25}

All major protocol modifications will be approved by the IRB, and participants will be reconsented as necessary. These changes will also be promptly communicated to *Trials* as updates after IRB approval.

#### Dissemination plans {31a}

At the conclusion of the trial, participants’ data will be analyzed at an individual (“N-of-1”) level. The PI will meet with participants to discuss their participation in the trial and present personal efficacy data. Exploratory analyses will also be conducted at a group level to determine overall efficacy results for the study sample. Findings will be submitted for publication to a peer-reviewed journal without publication restrictions.

## Discussion

We propose that the MCRCT design will allow us to efficiently determine the effects of MPH on symptoms of AD/ADRD at individual and group levels. We will also test the hypothesis that daily home-based brain games and other novel assessments will yield equivalent or better sensitivity to MPH treatment effects over repeated exposures. In the course of this work, we will appreciate the feasibility of this study design and the entirely remote, virtual setting based on participant tolerance, engagement, and compliance with this trial’s design and measures.

### Considerations for MCRCTs

One consequence of the MCRCT design is that it places increased demands on a single participant. Because each participant must undergo multiple treatment blocks, total time commitment for this trial type is generally longer than what is required for parallel-group RCTs. Daily, home-based assessments could also be potentially burdensome for participants, and we will pay special attention to assessment compliance and tolerability to determine future feasibility of these measures in AD/ADRD clinical trials. However, these additional burdens of MCRCTs may be offset by the guarantee of receiving active treatment, which is not the case in a parallel-group RCT where participants are at risk of being apportioned to the placebo arm. Another desirable aspect of the “N-of-1” MCRCT is that the observed treatment effect for a particular person can be estimated and shared with them at the conclusion of the trial, perhaps guiding subsequent clinical management.

MCRCTs may not be appropriate for some interventions, such as drugs with prolonged pharmacokinetics or delayed pharmacodynamic onset and cessation of action that would require longer treatment blocks. An intervention’s carryover effect must also be considered. While half-life of MPH is short, and the 2-week placebo duration should allow complete washout of any MPH pharmacological effect, we cannot exclude the possibility that carryover effects were not a factor. We do not expect carryover pharmacological effects from placebo, but carryover might differentially affect measurements made in a MPH treatment period that follows another MPH treatment period, with cumulative effects and/or longer taper. We will investigate various adjustments for carryover, such as discarding the first measurements in a treatment period on outcomes measured daily, to determine whether results are sensitive to carryover. These adjustments can allow for potential differential carryover by treatment regimen.

MCRCTs are considered most suitable for evaluating treatments of chronic, stable conditions rather than conditions that change over time. AD/ADRDs are progressive, so the effects of a given treatment may differ over the duration of the trial. However, AD/ADRDs are only very slowly progressive without major clinical changes over 6–12 months, so trials lasting a year or less can be conducted with minimal expected decline [3]. Nonetheless, it will be important to monitor such a possibility, even in a 4-month trial such as this.

The management and interpretation of repeated measures is an important element of MCRCT design. In repeated assessments of cognitive functioning, it is well-established that the occurrence of practice and learning effects stands to systematically influence observed performance on these repeat measures over time. This may be less of a concern in people with memory impairment than in other disorders [46–48] although still warrants consideration. Since the same standard neuropsychological assessments occur at nearly every study visit (in the present study, every 2 weeks), assessments with repeatable and equivalent forms, such as the RBANS, will be used to reduce practice and learning effects. Further, prior work has shown that performance on brain games can improve over time even in individuals with MCI or mild-stage dementia [49, 50]. However, this is likely a learning effect specific to game performance rather than a true, generalizable enhancement of cognition given that people tend to plateau in their performance with continued play. Thus, a lead-in time to achieve this plateau before entering the active treatment blocks of our study is an important feature. As long as performance is not at the ceiling or floor for a given measure, change in performance with treatment can be meaningfully interpreted.

This trial will also help us to determine the feasibility of running a virtual ADRD clinical trial. Although virtual administration is highly unconventional for neuropsychological assessments in clinical research trials, past research has shown that tele-neuropsychological assessments can be a valid and reliable alternative to in-office assessments in both cognitively healthy and impaired populations [51–53]. Virtual clinical trials may improve participation in research trials by decreasing the risk and burden of traveling to the research site and increase access to research for older adults who may not have otherwise been able to participate in a clinical trial. However, it does require access to a computer and an adequate level of technological literacy.

Finally, we use our MCRCT design to evaluate novel digital phenotyping tools (Fitbit actigraphy and sleep tracking, and speech production). These measures may respond to drug interventions or prove to be useful biomarkers for AD/ADRDs. Many of these assessments use low-cost, commercially available, non-invasive technologies. By developing these capabilities using such ubiquitous and largely passive technologies, we can enable more frequent assessments with lower participant burden and improve our estimates of treatment efficacy.

## Trial status

The current approved protocol is version 5.3 (August 31, 2020). The study has been approved since June of 2019 and is actively recruiting participants. As of publication, four participants have been randomized (three completed, one active, one early discontinuation). Recruitment is expected to continue until December 2020.

## Abbreviations

ABID: Agitated Behavior in Dementia Scale
AD: Alzheimer’s disease
ADCS-ADL-MCI: Alzheimer’s Disease Cooperative Study-Activities of Daily Living for Mild Cognitive Impairment
ADHD: Attention deficit/hyperactivity disorder
ADRDs: Alzheimer’s disease-related disorders
AES: Apathy Evaluation Scale
BAI: Beck Anxiety Inventory
BPM: Beats per minute
BVMT-R: Brief Visuospatial Memory Test-Revised
C-SSRS: Colombia-Suicide Severity Rating Scale
FDA: Food and Drug Administration
HIPAA: Health Insurance Portability and Accountability Act
ECG: Electrocardiogram
EEG: Electroencephalogram
FAQ: Functional Activities Questionnaire
GDS: Geriatric Depression Scale
IRB: Institutional Review Board
MCI: Mild cognitive impairment
MCRCT: Multi-crossover randomized controlled trial
MoCA: Montreal Cognitive Assessment
MPH: Methylphenidate
NPIQ: Neuropsychiatric Inventory-Questionnaire
PCP: Primary care physician
PI: Principal investigator
RBANS: Repeatable Battery for the Assessment of Neuropsychological Status
RCT: Randomized controlled trial
